# Evaluating the Prevalence and Risk Factors of Varicose Veins in Surgeons and Operating Room Staff at Buraidah Center Hospital

**DOI:** 10.7759/cureus.51706

**Published:** 2024-01-05

**Authors:** Bandar M Almutiri, Ahmed M Alshammari, Sarah B Alharbi, laila M Alamri, Aishah N Alsuhaibani, Rahaf S Alenazi, Ghayda K Alfarhan

**Affiliations:** 1 General Surgery, Buraidah Central Hospital, Buridah, SAU; 2 General Surgery, Saudi Commission for Health Specialties, Buraidah Central Hospital, Buraidah, SAU; 3 Medicine and Surgery, Qassim University, Buraidah, SAU; 4 College of Medicine and Surgery, Qassim University, Buraidah, SAU

**Keywords:** varicose vein surgery, varicose vein among medical staff, varicose vein prevalence, varicose vein risk factors, varicose vein

## Abstract

Background

Varicose veins (VVs), which are characterized by visible, convoluted veins in the lower limbs, are a prevalent disorder that afflicts a substantial portion of the population. The purpose of this cross-sectional study was to look at the prevalence and risk factors for VVs among surgeons and operating room personnel at Buraidah Central Hospital in Saudi Arabia. They usually become worse over time once they develop, which highlights the importance of early intervention and preventive actions.

Methodology

Data from 91 participants were collected from diverse healthcare professionals between August 2023 and September 2023 via an online questionnaire covering demographics, health, and occupational factors. The chi-square and Fisher's exact tests were employed to examine the correlation between these variables and the occurrence of VVs.

Results

The data analysis revealed that several specific factors displayed notable associations. Occupations as Surgical Physicians, OR Staff, or Nurse (p=0.009), the number of days worked in the operating room (p=0.040), the role in the operating room, especially those mainly standing (p=0.001), contraceptive pill usage (p=0.000), and vaginal delivery (p=0.037) displayed statistically significant relationships with VVs. In contrast, factors like gender, age group, ethnicity, family history of VVs, social status, smoking habits, exercise frequency, BMI, lifting heavy objects, and years in the field did not reveal substantial associations with VVs, as indicated by p-values exceeding 0.05.

Conclusion

The study identified a low VV diagnosis prevalence, with an equal distribution among male and female respondents. Key factors that contribute to the risk of developing VVs include the number of days worked in the operating room, the role in the operating room, a family history of VVs, contraceptive pill usage, and the method of delivery.

## Introduction

Varicose veins (VVs) in the lower limbs are the most common vascular concern, with potentially serious consequences, including mortality [1]. Worldwide, the reported prevalence of lower-limb VVs varies from 10% to 30% [1,2]. A dilation of 3 to 4 mm in the subcutaneous veins indicates the presence of VVs [3]. There are various types of vein disorders, such as reticular, telangiectasia, and trunk veins [4]. VVs can lead to aesthetic concerns and pain in the affected limbs, often necessitating surgery or other costly treatments that strain healthcare budgets [5]. The veins contain tiny valves that prevent blood from flowing backward. Malfunctioning of these valves allows blood to flow in reverse and accumulate in the veins, causing them to become more prominent and enlarged [6,7]. While the exact cause of VVs remains unclear, several exacerbating factors have been identified. Common risk factors include increasing age, being female, pregnancy, smoking, a family history of venous diseases, and being overweight [8-11]. Prolonged periods of standing at work significantly increase the likelihood of VV development and serve as a significant occupational risk factor [11].

Genetics, the deterioration of elastic tissue, and venous thrombosis within the venous wall are just a few factors that can lead to valve weakening and dysfunction [8,12]. The symptoms associated with VVs in the lower limbs may manifest in specific regions or affect the entire lower leg as a whole. Leg aches, heaviness, fatigue, and edema are also frequent symptoms, along with localized ones like discomfort, burning, and itching. Typically, standing for long periods makes the symptoms worse, and they get better when the patient sits and raises their legs [13,14]. VVs can lead to secondary issues such as skin pigmentation, atrophy blanche, edema, lipodermatosclerosis, venous ulcers, and varicose eczema. Direct complications of VVs include bleeding and thrombophlebitis [15]. Accurate diagnosis of the type and severity of venous insufficiencies, including VVs, often involves diagnostic tests such as a duplex Doppler test, phlebodynamometry, duplex ultrasound imaging, angioscopy, thermography, or capillaroscopy [16]. The Doppler approach for ultrasound examinations is increasingly frequently used and is thought to be useful for determining pathological reflexes, blood flow dynamics, and blockages. It is also thought to be helpful for early detection of the anatomical state through precise mapping of aberrant venous routes. Currently, there are a number of therapy options accessible. While some are more intrusive than others, such as endovenous laser therapy, surgical ligation, ultrasound-guided foam sclerotherapy, and radio-frequency ablation are examples of the former [5,17].

Internationally, numerous studies have estimated the prevalence of VVs in various populations. In Russia, a study conducted on chronic venous disease (CVD) found that 29% of individuals had primary VVs, with slightly higher rates in men (31.5%) than women (27.5%) [18]. In Brazil, a study found a 47.6% prevalence of VVs, with higher rates in non-pregnant women (50.9%) compared to men (37.9%) [19]. Additionally, research conducted in western Jerusalem estimated that 10% of men and 29% of women in the population had VVs. The prevalence rises with age and gender, reaching 54% in women between the ages of 65 and 74 and 39% in men over the age of 75 [20]. According to a study conducted in Jeddah, Dammam, and Makkah in Saudi Arabia, 66% of females in the general population are affected by VVs [21].

Most of the research on VVs has primarily focused on treatment and therapeutic outcomes, with studies mainly examining general risk factors in diagnosed VV patients. There is a need for more studies that investigate occupational risk factors in the broader population. A significant portion of studies that have delved into occupational risk factors related to VVs have centered on the effects of prolonged standing during work. An inpatient cohort study reported by Tuschen et al. [22] determined that, after adjusting for age and smoking status, the risk ratio for VV prevalence associated with prolonged standing was 1.85 (95% CI = 1.33-2.36) for men and 2.63 (95% CI = 2.25-3.02) for women. Likewise, a study in Korea revealed a strong link between prolonged standing at work and the incidence of VV, a relationship that persisted even among individuals of a younger age and those with less work experience. The estimated prevalence of VV in this context was approximately 16.18% [10]. However, a study among physicians in Taiwan suggested that long hours of standing did not significantly impact the development of VV, both among physicians and non-physicians [23].

In Saudi Arabia, there has been limited research on the prevalence of VVs in both genders of the population and the relationship between VVs and occupation type, duration of standing or sitting at work, and lifestyle factors like smoking and exercise [16,17]. This study aims to investigate the demographic and occupational factors that predict VV diagnosis among surgeons and OR staff at Buraidah Center Hospital. It also seeks to assess the prevalence and identify the key risk factors for VVs among these populations. To understand the correlation between prolonged standing and sitting durations and shift lengths with the frequency of this condition, it is essential to gather information on the prevalence of VVs in various hospital departments. 

## Materials and methods

This was a cross-sectional observational study that primarily depended on a self-administered questionnaire for data collection. The study was conducted in Buraidah Central Hospital, Qassim Region, in Saudi Arabia. It is conducted from August 2023 to November 2023. The data collection period has been completed in September 2023. Our study focused on all surgeons with different specialties, anesthetists, nurses, radiology technicians, and other staff at Buraidah Central Hospital in the Qassim Region, Saudi Arabia. An online Google form link to participate in the research was shared, with no prior sensitization provided and no follow-up attempts. Inclusion criteria included all individuals who met the male and female surgeons with different specialties and anesthetists, nurses, radiology technicians, and other OR staff were included. Exclusion criteria included doctors other than surgery specialties, nurses with medical specialists and wards, and other hospital staff.

Our samples were 91 self-administered online questionnaires. The data were obtained from an online questionnaire created based on a previously published questionnaire [24]. The questionnaire was an open survey that consisted of multiple sections. The demographic section (gender, age, ethnicity, social status, department), behavioral section (exercising or smoking), physical section (BMI), and work-related variables section (lifting heavy objects, playing sports, or performing physical activities) were all evaluated based on the subjective responses provided by the participants. Health-related sections (family history of deep vein thrombosis, VVs, coronary artery disease, hypertension, diabetes, rheumatic arthritis, chronic constipation, past surgical history, and occupational injuries to the lower limbs) for females (hormonal therapy, contraceptive use, number of gravidas, parities, type of delivery, menopausal status) were also included. Additionally, we incorporated work-related questions in the questionnaire, including the duration of experience in the field, the frequency of weekly shifts, any prior diagnoses of VVs, and the amount of time spent standing and sitting during work. The questionnaires were distributed to participants through both electronic mail and social media platforms. Prior to their participation, individuals voluntarily expressed their verbal agreement and provided informed consent to be part of the research study. To safeguard the data, the questionnaire responses were recorded in an Excel spreadsheet and stored on a securely locked and password-protected laptop. 

This study received ethical approval from the Regional Research Ethics Committee, Qassim Province, Registered at National Committee of Bio & Med. Ethics (NCBE) Registration No. H-04-Q-001. All gathered data was treated with confidentiality, and access was limited to the researchers involved. The publication exclusively presented a statistical summary and did not contain any personal information to ensure privacy and data protection.

All data were analyzed using IBM SPSS Statistics for Windows, Version 23 (Released 2015; IBM Corp., Armonk, New York, United States). Continuous data were summarized with the mean, mode, and standard deviation, while categorical data were described using numbers, percentages, and frequencies in descriptive statistics. To evaluate the relationships between variables, chi-square tests and t-tests were employed. A significance level of p < 0.05 was considered to indicate statistical significance.

## Results

This study comprised a total of 91 participants, with the gender distribution showing that the majority of participants, 53 (58.2%), were male. In contrast, 38 (41.8%) were female, with the majority of the age group between 41 and 50 years (36.3%). Most of the participants, numbering 64 (70.3%), identified as Asian, with 20 (22%) classifying themselves as African. Regarding marital status, 70 (76.9%) of the participants were married, 20 (22%) were single, and 1 (1. 1%) were widowed. In terms of workplace distribution, slightly more than half, 61 (67.0%), were physicians, and 26 (28.6%) were nurses. When it came to smoking habits, 76 (83.5%) were nonsmokers, and 15 (16.5%) were smokers. In terms of physical activity, nearly half of the participants, 54 (59.3%), engaged in physical exercise or sports four times a week or less, while 30 (33.0%) did not engage in these activities. Most of the participant's BMI, 31 (34. 1%), lay between 25 and 29.9, while 27 (29.7) in between 30 and 34.9. Almost an equal number of participants reported either lifting heavy objects 44 (48.4%) or not 47 (51.6%). Forty-seven participants (51.6%) spent 10 years or more working in the field, 11 (12. 1%) worked for 5-9 years, 22 (24.2%) worked for 1-4 years, and 11 (12. 1%) worked from less than one year, and mainly majority of participants, 57 (62.6%), standing during their work, 2 (2.2%) were sitting, and 32 (35.2%) were in both positions. The maximum number of hours participants 47(51.6%) spent sitting was 1-2 hours, and 37 (40.7%) participants spent 5-6 hours while standing. The study found that 12 (13.2%) of the respondents were diagnosed with VVs, while the majority, 79 (86.8%), did not have this condition. The individuals had a comparatively low prevalence (46.2%) of illnesses such as deep vein thrombosis, coronary artery disease, hypertension, diabetes, rheumatoid arthritis, chronic constipation, past surgical history to lower extremities and past trauma history to the lower extremities, while the majority of the participants (53.8%) responded no for these health conditions. Additional details on contraceptive use, menopause status, the number of children, and the mode of delivery were provided by the female respondents. With the aid of these extensive data, participants in the study's demographics, lifestyles, and health features can be fully comprehended (Table 1). The age and BMI information was indeed obtained from the participants. However, in the Results section, we only presented the data in age categories for simplicity purposes. The standard deviation for age and height was not included in the table, but the BMI values were calculated using the participants' self-reported height and weight measurements.

**Table 1:** Demographic characteristics of participants working at Buraidah Center Hospital

Variables	Frequency (n= 91)	Percentage (%)
Gender	-	-
Female	38	41.8
Male	53	58.2
Age group	-	-
25-30 years	18	19.8
31-40 years	24	26.4
41-50 years	33	36.3
>50 years	16	17.6
Ethnicity	-	-
Arabian	1	1. 1
Asian	64	70.3
African	20	22.0
Other	6	6.6
Social status	-	-
Married	70	76.9
Single	20	22.0
Widow/Widowed	1	1. 1
Divorced	0	0
Are you working as	-	-
Surgical physicians	55	60.4
OR Staff	36	39.6
What is your job in the operation room	-	-
Anesthesia technician	3	3.3
Nurse	26	28.6
OR technician	1	1. 1
Physicians	61	67.0
Are you a smoker	-	-
No	76	83.5
Yes	15	16.5
How often do you perform physical or play sports exercise per week?	-	-
Five times or less	7	7.7
Four times or less	54	59.3
No	30	33.0
BMI	-	-
<18.5	5	5.5
18.5-24.9	7	7.7
25-29.9	31	34. 1
30-34.9	27	29.7
>35	21	23. 1
Lifting heavy objectives	-	-
No	47	51.6
Yes	44	48.4
How many years have you spent on work in the field?	-	-
Less than one year	11	12. 1
1-4 years	22	24.2
5-9 years	11	12. 1
Ten years or more	47	51.6
How many days per week you work on OR?	-	-
1-2 days	38	41.8
3-4 days	18	19.8
5-7 days	35	38.5
What is your role in the OR?	-	-
Mainly sitting	2	2.2
Mainly standing	57	62.6
Both	32	35.2
How many hours do you sit during your work per day?	-	-
1-2 hours	47	51.6
3-4 hours	30	33.0
5-6 hours	7	7.7
More than 6 hours	7	7.7
How many hours standing during your work per day?	-	-
1-2 hours	7	7.7
3-4 hours	29	31.9
5-6 hours	37	40.7
More than 6 hours	18	19.8
Do you have family history of varicose veins?	-	-
No	73	80.2
Yes	18	19.8
Have you ever been diagnosed with varicose veins?	-	-
No	79	86.8
Yes	12	13.2
Do you have deep vein thrombosis?	-	-
No	91	100
Yes	0	0
Do you have coronary artery disease?	-	-
No	89	97.8
Yes	2	2.2
Do you have hypertension?	-	-
No	80	87.9
Yes	11	12. 1
Do you have diabetes?	-	-
No	86	94.5
Yes	5	5.5
Do you have rheumatoid arthritis?	-	-
No	88	96.7
Yes	3	3.3
Do you have chronic constipation?	-	-
No	79	86.8
Yes	12	13.2
Do you have a past surgical history of lower extremities?	-	-
No	87	95.6
Yes	4	4.4
Do you have past trauma history to the lower extremities?	-	-
No	86	94.5
Yes	5	5.5
Are you on contraceptive pills (only for female respondents)?	-	-
No	31	81.5
Yes	7	18.4
Menopause	-	-
No	38	100
Yes	0	0.0
How many children do you have	-	-
None	14	36.8
1 child	6	15.7
2 children	10	26.3
3 children	5	13. 1
4 children	2	5.2
More than four children	1	2.6
Type of delivery	-	-
None	14	36.8
Vaginal	16	42. 1
C-section	7	18.4
Both	1	2.6

Table 2 provides a thorough analysis of the correlation between various factors and the occurrence of VVs within the cohort of 91 participants in the study. Notably, the data revealed that in terms of gender (p=0.547), age group (p=0.611), ethnicity (p= 0.372), and social status (p= 0. 126) no statistically significant associations with VVs were observed, as evidenced by p-values exceeding 0.05. However, some key associations stand out. Working as a surgical physician or OR staff, as well as being a nurse (p= 0.009), showed a significant connection to VVs, highlighting potential occupational influences. Similarly, the number of days worked in the operating room (p= 0.040) exhibits a significant link, suggesting that increased exposure to specific workplace conditions may contribute to VVs. The occupation in the operating room, particularly for those individuals predominantly standing (p = 0.001), showed a significant association with VVs. Additionally, variables such as a family history of VVs (p= 0.056), contraceptive pill usage (p= 0.000), and the type of delivery, notably vaginal (p= 0.037), showed statistically significant relationships with the presence of VVs. Other health-related conditions and lifestyle factors like smoking habits (p= 0.205), physical exercise frequency (p= 0.995), BMI (p= 0.562), lifting heavy objects (p= 0.760), and years in the field (p= 0.565) did not exhibit any significant associations with VVs. In summary, Table 2 offers valuable insights into the factors associated with VVs within the studied population, shedding light on both potential risk factors and areas with no evident connections.

**Table 2:** Comparison of categorical and continuous variables of participants with and without varicose veins

Variables	With varicose vein frequency (n=12)	p-value	Without varicose vein frequency (n=79)	p-value
Gender	-	0.035	-	0.547
Female	6		32	
Male	6		47	
Age group	-	0.054	-	0.611
25-30 years	4		14	
31-40 years	2		22	
41-50 years	4		29	
>50 years	2		14	
Ethnicity	-	0.592	-	0.372
Arabian	0		1	
Asian	11		53	
African	1		19	
Other	0		6	
Social status	-	0.232	-	0. 126
Married	12		58	
Single	0		20	
Widow/Widowed	0		1	
Divorced	0		0	
Are you working as	-	0.009	-	0. 140
Surgical physicians	3		52	
OR Staff	9		27	
What is your job in the operation room	-	0.057	-	0.040
Anesthesia technician	2		1	
Nurse	2		24	
OR technician	0		1	
physicians	8		53	
Are you smoker	-	0.002	-	0.205
No	12		64	
Yes	0		15	
How often do you perform physical or play sports exercise per week	-	0.933	-	0.995
Five times or less	1		6	
Four times or less	7		47	
No	4		26	
BMI	-	0.77	-	0.562
<18.5	0		5	
18.5-24.9	0		7	
25-29.9	5		26	
30-34.9	5		22	
>35	2		19	
Lifting heavy objectives	-	0. 151	-	0.760
No	7		39	
Yes	5		5	
How many years you spend on work in the field	-	0.210	-	0.565
Less than one year	2		9	
1-4 years	3		19	
5-9 years	0		11	
Ten years or more	7		40	
How many days per week you work on OR	-	0.052	-	0.015
1-2 days	3		35	
3-4 days	0		18	
5-7 days	9		26	
What is your role in the OR	-	0.042	-	0.001
Mainly sitting	0		2	
Mainly standing	2		55	
Both	10		22	
How many hours sitting during your work per day	-	0.022	-	0.435
1-2 hours	8		39	
3-4 hours	4		36	
5-6 hours	0		7	
more than 6 hours	0		7	
How many hours standing during your work per day	-	0.204	-	0.567
1-2 hours	2		5	
3-4 hours	3		26	
5-6 hours	4		33	
more than 6 hours	3		15	
Do you have Family history of Varicose Veins	-	0.056	-	0.536
No	7		66	
Yes	5		13	
Do you have Deep vein thrombosis	-	0.832	-	0.577
No	12		79	
Yes	0		0	
Do you have coronary artery disease	-	0.678	-	1.000
No	12		77	
Yes	0		2	
Do you have hypertension	-	0.673	-	1.000
No	11		69	
Yes	1		10	
Do you have diabetes	-	0.373	-	0.515
No	11		75	
Yes	1		4	
Do you have rheumatoid arthritis	-	0. 160	-	1.000
No	12		76	
Yes	0		3	
Do you have chronic constipation	-	0.468	-	0.656
No	10		69	
Yes	2		10	
Do you have a Past surgical history of lower extremities	-	0.098	-	1.000
No	12		75	
Yes	0		4	
Do you have past trauma history to the lower extremities	-	0.059	-	1.000
No	12		74	
Yes	0		5	
Are you on Contraceptive pills	-	0.567	-	0.000
No	1		30	
Yes	5		2	
Menopause	-	0.932	-	0.547
No	6		32	
Yes	0		0	
How many children do you have	-	0.521	-	0.378
None	1		13	
1 child	1		5	
2 child	1		9	
3 child	2		3	
4 child	1		1	
More than 4 child	0		1	
Type of delivery	-	0.567	-	0.037
None	1		13	
Vaginal	4		12	
C-section	0		7	
Both	1		0	

## Discussion

Lower-limb varicose veins, or VVs, manifest as twisted and visible veins in the lower extremities due to blood reflux within the veins. This reflux occurs as a result of the dilation of vein walls and the failure of valves in superficial veins to function properly. This condition is quite common, affecting a significant portion of the general population, with estimates ranging from 10% to 30% [13]. Importantly, once VVs develop, they do not spontaneously improve, and their associated symptoms tend to worsen with time. Therefore, it is crucial to actively address VVs during their early stages. Even more significantly, it is essential to take preventive measures against their risk factors before symptoms become apparent. This discussion delves into the key findings and implications of a study aimed at understanding the prevalence and risk factors of VVs among surgeons and OR staff working in Buraidah Center Hospital. In a cross-sectional survey organized in Buraidah Center Hospital, Saudi Arabia, involving 91 participants, the gender distribution revealed 38 females (41.8%) and 53 males (58.2%). Previous research has reported a wide range of VV prevalence, varying from as low as 2% to as high as 56% in males and from 1% to 73% in females [24]. Out of the total participants, 12 individuals (13.2%) had been diagnosed with VVs, with male (6.5%) and female (6.5%) respondents exhibiting an equal prevalence of VVs. Notably, Sharif Nia's research has suggested a gender-based risk for VV, indicating that females are more susceptible to this condition [10,25].

In this study, the various risk factors associated with VVs were working as surgical physicians or OR staff, as well as being a nurse, the number of days worked in the operating room, role in the operating room, family history of VVs, contraceptive pill usage, and the type of delivery. The data predicted that gender, age group, ethnicity, social status, smoking habits, physical exercise frequency, BMI, lifting heavy objects, hours standing per day, hours sitting per day, and years in the field did not show any statistically significant associations with VVs. These variables have consistently emerged as significant risk factors for VVs in the majority of prior research studies [5,21,25]. Hours standing per day and hour sitting per day were statistically significant in our study. Also, the number of days worked in the operating room (p= 0.040) exhibits a significant link, suggesting that increased exposure to specific- workplace conditions may contribute to VVs. The role in the operating room, especially those mainly standing (p= 0.001), is significantly associated with VVs. Kohno et al. conducted a study involving adults aged 45 or older and found that prolonged standing at work considerably raised the risk of VVs [26]. Chen and Guo's research on hairdressers and VVs demonstrated that individuals with VVs had longer work histories (30.5 vs. 24.0 years), were older (49.3 vs. 44.7 years), and spent more hours working in a standing position each month (213.9 vs. 176.0 hours) in comparison to those without VVs [27].

Among studies investigating occupational factors in the population, Kohno et al. focused on adults aged 45 and older [26]. They found that prolonged standing position at work significantly increased the risk of developing VVs (odds ratio [OR] = 3.42, 95% CI = 1.07-10.89). This suggests that extended periods of standing are a risk factor for VV incidence among the general population, regardless of their specific occupation. Other studies examining VV risk in specific occupational groups, such as Chen et al. [27] among hairdressers and Sharif et al. [25] among nurses, also reported an elevated risk of VVs associated with longer hours of standing work. These findings reinforce the link between VV incidence and occupations involving extended periods of standing.

Lifting heavy objects and years of working were not the risk factors for VV in Buraidah Center Hospital. The results found in the studies conducted by AlBader et al. and Chen are inconsistent with our findings, indicating a significant association between greater years of work experience and the presence of VVs [16,25]. However, Ali et al. suggested that years of working was not the risk factor for VVs [24]. Furthermore, our data revealed a significant correlation between VVs and a family history of the condition, which is consistent with the findings of studies by Laurikka et al. and AlBader et al. [16,28]. The risk of inheriting VVs was substantial, with children having a 90% chance if both parents had the condition, 25% if one parent was affected (for men), and 62% (for women). The risk dropped to 20% if neither parent had VVs [29].

In our study, we did not observe a significant correlation between VVs and hypertension, which contrasts with other studies in the literature that have linked VV to high blood volumes and dilated vessels [24,29]. However, AlBader et al. also showed an insignificant association between VVs and hypertension [16]. Similarly, we found no association between diabetes, thrombosis, coronary artery disease, rheumatoid arthritis, and constipation with VV, which deviates from previous research indicating a connection between these conditions [24,30]. However, these factors were also considered insignificant by some other studies [6,16]. Additionally, it is worth noting that a significant proportion of our participants were nonsmokers, and they fell within the middle-aged category with a typical BMI range of 25-29.9. These combined factors could collectively impact the development of VVs, highlighting the significance of occupational factors as stronger predictors for VV formation in this specific population.

Results found that females who gave birth vaginally were at a higher risk of developing VVs compared to those who delivered via C-section. Moreover, females with children faced a greater risk of VVs than those without. This increased risk during pregnancy can be attributed to the elevated levels of progesterone and the expansion of the uterine wall, both of which lead to an increase in blood volume and can potentially result in valve failure, contributing to VV development. This observation is consistent with the research of Jawien et al., who documented a higher prevalence of VVs in women when compared to men [31]. Furthermore, the number of pregnancies, especially exceeding two pregnancies, emerged as a significant distinguishing factor between women with and without VVs.

It is essential to acknowledge the limitations of this study. As this study is cross-sectional and conducted at a specific point in time, it is crucial to acknowledge that causality cannot be conclusively established between the identified risk factors and the onset of VVs. Additionally, the study was conducted within a specific hospital setting, which may only be generalizable to some healthcare facilities. Further research is needed to explore the complex interplay of occupational and genetic factors in VV development.

## Conclusions

This study provides valuable insights into the incidence and risk factors of VVs in surgeons and OR staff at Buraidah Central Hospital. The results found that a low prevalence (13.2%) of respondents had been diagnosed with VVs, with male and female respondents exhibiting an equal prevalence of VVs. Risk factors associated with VVs were the number of days worked in the operating room, role in the operating room, family history of VVs, contraceptive pill usage, and the type of delivery. The findings highlight the need for healthcare institutions to address the well-being of their employees and implement strategies to mitigate the risk of VVs. 
